# Intelligent Agent-Based Intrusion Detection System Using Enhanced Multiclass SVM

**DOI:** 10.1155/2012/850259

**Published:** 2012-09-27

**Authors:** S. Ganapathy, P. Yogesh, A. Kannan

**Affiliations:** Department of Information Science and Technology, Faculty of Information and Communication Engineering, Anna University, Guindy, Chennai 600025, India

## Abstract

Intrusion detection systems were used in the past along with various techniques to detect intrusions in networks effectively. However, most of these systems are able to detect the intruders only with high false alarm rate. In this paper, we propose a new intelligent agent-based intrusion detection model for mobile ad hoc networks using a combination of attribute selection, outlier detection, and enhanced multiclass SVM classification methods. For this purpose, an effective preprocessing technique is proposed that improves the detection accuracy and reduces the processing time. Moreover, two new algorithms, namely, an Intelligent Agent Weighted Distance Outlier Detection algorithm and an Intelligent Agent-based Enhanced Multiclass Support Vector Machine algorithm are proposed for detecting the intruders in a distributed database environment that uses intelligent agents for trust management and coordination in transaction processing. The experimental results of the proposed model show that this system detects anomalies with low false alarm rate and high-detection rate when tested with KDD Cup 99 data set.

## 1. Introduction

Mobile ad hoc networks (MANETs) consist of mobile nodes that work independently without an infrastructure. They are useful in application areas like disaster management emergency and rescue operations where it is not possible to have well-defined infrastructure. MANETs are characterized by its great flexibility. However, MANET's inherent vulnerability increases their security risks. Though MANETs are dynamic and cooperative in nature, it needs efficient and effective security mechanisms to safeguard the mobile nodes. Intrusion detection and prevention are primary mechanisms to reduce possible intrusions. Intrusion detection using classification algorithms effectively discriminates “normal” behavior from “abnormal” behavior. Therefore, intrusion detection and prevention system can be used as a secondary mechanism of defense in any wireless environment and mobile databases so that it can be a part of the reliable communication in MANETs [1].

Intrusion detection systems (IDS) play a major role in providing security to networks. In this paper, we introduce a new intelligent agent-based intrusion detection model for securing the mobile ad hoc networks. The main function of the proposed intrusion detection system is to monitor the computer system and network in order to find the intrusion activities in the system. In such system, attacks are divided into two categories, namely, host-based attacks and network-based attacks. Hence, IDSs are also classified into two categories, namely, network intrusion detection system (NIDS) and host-based intrusion detection system (HIDS). Another way to classify IDSs is with respect to the way in which they detect intrusions. According to this classification, there are two categories of intrusions, namely, anomaly-based intrusion and misuse-based intrusion. Anomaly-based IDS is able to identify malicious traffic based on the deviations from the preestablished normal network traffic patterns. In misuse-based detection, network traffic is examined for preconfigured and predetermined attack patterns.

An intelligent agent is a program module that functions continuously in a particular environment senses it and acts based on the environmental conditions. Therefore, it is able to carry out activities in a flexible and intelligent manner to be responsive to changes in the environment. Moreover, such an agent is able to learn from its experiences. Since the agent is autonomous, it takes actions based on its built-in knowledge and its past experiences stored in the form of rules. Intelligent agents can be classified into four categories, namely, simple reflex agents, agents that keep track of the world using learning, goal-based agents, and utility-based agents. The simple reflex agents perceive the input from their environment and interpret it to a state that matches their rules. The agents that keep track of the world maintain an internal state of past inputs because their actions need to occur in correlation with the past states and the new state. The goal-based agents need to know some information about their goals because the percepts (impression of the object obtained using the senses) do not provide enough information to determine the action to be taken. Sometimes knowing the goals are not sufficient for the agents to take the right action, especially when there are conflicting goals. As a result, the utility-based agents map the percept states into numbers that determine how closely the goals were achieved [2]. Intelligent agents, as a modern artificial intelligence concept, are now widely deployed in various software systems [3].

Outlier detection is an important activity in many safety critical environments [4], where the outlier indicates the abnormal running conditions from which major performance deprivation may result. An outlier is an IDS that denotes an anomaly node in a network and also the anomaly data in distributed systems. An outlier pinpoints an intruder inside a system with malicious intentions quickly. Outlier detection accomplishes this by analyzing and comparing the time series data regarding the usage statistics. Data preprocessing plays an important role in any intrusion detection system since it is used to produce a filtered data to intrusion detection system. Therefore, data preprocessing algorithms help to improve the performance as well as detection accuracy and to reduce the training time.

In this paper, a new intelligent agent-based intrusion detection model called Intelligent Agent based Feature Selected Hybrid Classifier (IAFSHC) for detecting the intruders in wireless ad hoc networks has been proposed for securing the networks. For this purpose, a combination of an intelligent agent-based weighted outlier detection algorithm and an intelligent agent-based enhanced multiclass SVM algorithm for classification have been proposed in order to classify the attacks effectively. Moreover, we propose a new intelligent agent-based attribute selection algorithm to improve the performance. Even though various types of attacks happen in the internet scenario, we have focused mainly on the effective detection of DDoS attacks in this paper. The intelligent agents proposed in this paper act intelligently for classifying the intruders based on their behaviors. The major application of this work is the provision of secure environment in serious situation such as battle fields, earth quake, zones, and places where natural disasters like storm occur. 

## 2. Motivation for the Proposed Work

The rapid developments in the usage of wireless and mobile ad hoc networks have changed the scope of network security. The nature of mobility in such networks leads to new vulnerabilities that do not exist in a fixed wired network. Even though, many security mechanisms have been proposed in the past to protect MANETs from vulnerabilities, most of the existing security measures and methods turn out to be ineffective in the current internet scenario due to the introduction of new type of attacks. Therefore, the traditional way of protecting networks with firewalls and encryption software is no longer sufficient. Hence, it is necessary to develop new mechanisms to protect the wireless networks and mobile computing applications effectively.

There are various reasons why wireless ad hoc networks are at risk from a security point of view. In traditional wireless networks, mobile nodes associate themselves with an access point, which in turn are connected to other networking devices such as gateways or name servers that are responsible for handling the network management functions. On the other hand, ad hoc networks do not have such a centralized access point for communication and control. Moreover, the absence of centralized control makes it difficult for the nodes to know the trust values of other nodes participating in the communication. Since there is no ground for a prior classification of nodes based on a trust value, all nodes in MANET are required to cooperate in supporting the network operations and hence no prior security association can be assumed for all the participating nodes. In such a scenario, the freely roaming nodes form transient associations with their neighbors by joining and leaving subdomains independently with and without notice. Due to all these factors in ad hoc networks, the wireless links between nodes are highly susceptible to link attacks, which include passive eavesdropping, active interfering and leakage of secret information, data tampering, impersonation, message replay, message distortion, and denial of service (DoS). Hence, it is necessary to secure the ad hoc network from attacks by intruders using effective intrusion detection techniques.

Moreover, the dramatic increase in attacks in the internet results in damage of most sensitive information. Since, attacks are becoming more and more complex and attackers focus on new vulnerabilities, the existing intrusion detection techniques are not able to detect and handle the new types of attacks. Therefore, an intelligent intrusion detection technique which can handle both known and unknown types of attacks must be developed. 

## 3. Literature Survey

There are many works that are presented in the literature that discuss about techniques for intrusion detection. Among them, Bakar et al. [7] proposed a new agent-based approach for intrusion detection using rough-set-based classification technique. This technique generates rules from a large database and has mechanisms through rough sets to handle noise and uncertainty in data. However, producing a rough classification model or rough classifier is computationally expensive, especially in its reduct computation phase. Tweedale et al. [8] proposed a neural network (NN) based multiagent classifier system (MACS) using the trust, negotiation, and communication (TNC) reasoning model for intrusion detection. The main contribution of their work is proposed in a trust measurement method based on cognition and rejection rates. They proposed two types of agents implementing their system with respect to trust management and classification. Wang and Chiang [9] have proposed a cluster validity measure with outlier detection and cluster merging algorithms for providing a support vector clustering algorithm. This algorithm is capable of identifying the ideal cluster numbers with compact and smooth arbitrary-shaped cluster contours for increasing robustness of outliers and noises.

There are many classification algorithms that are found in the literature. For example, an algorithm called tree-structured multiclass SVM has been proposed by Mulay et al. [10] for classifying data effectively. Their paper proposed a decision tree-based algorithm to construct a multiclass IDS which is used to improve the training time, testing time, and accuracy of IDS. Support vector machine can easily achieve the high-detection accuracy for every attack instances of data. By using the feature ranking method can get better accuracy for DoS attacks [11]. Multiclass SVM algorithm can implement or be used for intrusion detection system. Integration of decision tree model and SVM model gives better results than the individual models [10]. Farid et al. [12] proposed a new learning approach for intrusion detection. It performs data reduction by the help of selecting the important subset of attributes.

Attribute reduction is very useful to delete less important attributes by applying heuristic functions. A redundant algorithm for attribute selection was proposed by Yang et al. [13]. Moreover, Teng et al. [14] introduced an efficient attribute reduction algorithm which simplified the consistent decision table. They have shown that the knowledge reduction is feasible and effective in reducing the attributes which is suitable to classify a huge data set. Wang et al. [15] proposed four different methods of attribute normalization to preprocess the data for anomaly intrusion detection. These methods are useful for providing fast classification.

There are many works in the literature that discuss about intrusion detection, classification, and outlier detection for intrusion detection. Among them, Angiulli et al. [4] have proposed a distance-based outlier detection method, which is used to find the top outliers in an unlabeled data set and to provide a subset of it, called the outlier detection solving agent. This solving agent can investigate the accuracy effectively based on outliers. A novel intrusion detection method by combining two anomaly methods, namely, conformal predictor K-Nearest Neighbor (KNN) and distance-based outlier detection (CPDOD) algorithm was proposed by Abdel-Fattah et al. [16] to detect anomalies with low false alarm rate and high-detection rate. An instance-based anomaly detection algorithm proposed by Teng [17] to reduce the number of distance computation of time series. In this algorithm abnormal time series is efficiently detected and also provides better accuracy than basic outlier detection algorithms.

In the past, we have proposed [6] a new-weighted-based outlier detection algorithm called weighted distance based outlier detection (WDBOD) algorithm for effective separation of outliers. In another work, we introduced a new intrusion detection model [1] for detecting the attackers in MANETs using a combination of enhanced multiclass SVM and WDBOD. In this paper, this combined model is named as feature selected hybrid classification (FSHC) model. In this paper, we propose an intelligent agent-based frame work for intrusion detection using preprocessing, outlier detection and enhanced multiclass SVM where all the decision activities are supported by intelligent agent and the trust values of the participating nodes which are maintained by these intelligent agents. Comparing with all the works in the literature the IDS proposed in this paper is different in many ways. First, we propose a new intelligent agent-based preprocessing algorithm to detect the attackers. Second, we propose an intelligent agent-based weighted outlier detection algorithm for finding relevant data. Third, we use an intelligent agent-based enhanced MSVM algorithm for classification. Fourth, we focus on DDoS attacks which are the most important among the different types of attacks. Finally, we use the KDD cup data set for carrying the experiments on providing security to MANETs.

## 4. System Architecture

**Figure 1:**
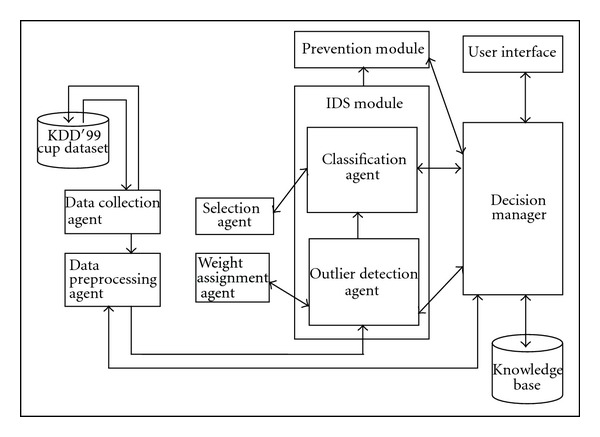
System architecture.

The architecture of the system proposed in this work consists of seven major components, namely, data collection agent, data Preprocessing module, intrusion detection system module and prevention module, user interface, decision manager, and knowledge base as shown in Figure 1.

### 4.1. Data Collection Agent

The Data collection agent collects the network data from the network layer. This data are sent to the preprocessing module for preprocessing the data.

### 4.2. Data Preprocessing Agent

The data preprocessing agent uses a preprocessing technique called attribute selection algorithm for effective preprocessing. In this technique, the agent selects only the valuable attributes from the data set using projection. Moreover, data cleaning, data integration, and data transformation are carried out for performing effective preprocessing.

### 4.3. Intrusion Detection Module

The intrusion detection module detects the intruders from the given data using intelligent agent-based weighted distance detection algorithm and intelligent agent-based enhanced multiclass support vector machine algorithm. The intrusion detection module distinguishes the intruders from normal users using an outlier detection algorithm which is combined with SVM for obtaining better classification accuracy.

#### 4.3.1. Outlier Detection Agent

This intelligent agent uses a newly proposed weighted-distance-based outlier detection algorithm where the agent uses an outlier factor to determine the outlier of a point in the feature set.

The agent is used to improve the detection accuracy and it gets valuable suggestions from the weight assignment agent.

**Weight Assignment Agent**

This agent assigns the correct weight for all features in the dataset. Moreover, it forwards the valuable suggestions to the outlier detection agent. This agent is helpful to assign the correct weight for all features and also to improve the classification accuracy.

#### 4.3.2. Classification Agent

This intelligent agent uses a newly proposed intelligent agent-based enhanced support vector machine algorithm where the agent effectively classifies the data using a suitable distance measurement formula.

**Selection Agent**

This agent selects the effective distance measure for classification. The classification agent gets the distance measurement formula which is selected by the selection agent.

### 4.4. Prevention Module

The prevention module is used to prevent the attacks detected by the intrusion detection module. 

### 4.5. Decision Manager

The decision manager monitors the overall process of this proposed system. The decision manager makes the decision about the classification and prevention activities by the help of rules present in the knowledge base. Moreover, it decides the activities of preprocessing. The user interface is provided in the system for interacting with the decision manager through queries.

**Knowledge Base**

The knowledge base contains rules for making decisions about the attribute selection, outlier detection, classification, and prevention. It also provides replies to the user queries and to perform effective decision making.

### 4.6. User Interface

Users can interact with the system through this user interface. User interface gives the request to the decision manager and gets the result.

## 5. Proposed Intrusion Detection Model

In this paper, we propose a new intrusion detection model using the combination of outlier detection and classification techniques. The classification is performed by a combination of intelligent agent-based weighted distance based outlier detection (IAWDBOD) and intelligent agent-based enhanced multiclass support vector machine (IAEMSVM) algorithms. In addition, we propose an attribute selection algorithm called intelligent agent-based attribute selection algorithm (IAASA).

### 5.1. Intelligent Agent-Based Attribute Selection Algorithm (IAASA)

The attribute selection algorithm [5] is used by the preprocessing agent where the information gain ratio for attribute selection is defined as follows. Let *D* be the data set which is listed into *n* number of classes. Let *F*_*i*_ be the attributes with maximum number of nonzero values chosen by the agent

(1) $$\begin{array}{c} \text{Info}\left(D\right)=-\left[\frac{\text{freq}\left(C_{j},D\right)}{\left|D\right|}\right]\log_{2}\left[\frac{\text{freq}\left(C_{j},D\right)}{\left|D\right|}\right],\\ \text{Info}\left(F\right)=\left[\frac{\left|F_{i}\right|}{\left|F\right|}\right]*\text{info}\left(F_{i}\right),\\ \text{IG}R\left(A_{i}\right)=\left[\frac{\text{Info}\left(D\right)-\text{Info}\left(F\right)}{\text{Info}\left(D\right)+\text{Info}\left(F\right)}\right]*100. \end{array}$$

*Input.* Set of 41 features from KDD cup Data Set

*Output.* Reduced set of features *R*.

Steps of the algorithm: the intelligent agent performs the following steps.

 
*Step  1*. Calculate the information gain for each attribute *A*_*i*_ *εD* using (3).
 
*Step  2*. Choose an attribute *A*_*i*_ from *D* with the maximum information gain value.
 
*Step  3*. Split the data set *D* into subdatasets {*D*_1_, *D*_2_,…*D*_*n*_} depending on the attribute values of *A*_*i*_ where *C*_*j*_ stands for *j*th attribute of class *C*.
 
*Step  4*. Find all the attributes whose information gain ratio >threshold.
 
*Step  5*. Store the selected attributes in the set *R* and output it.

### 5.2. Multiclass Support Vector Machine

The multiclass support vector machine (MSVM) algorithm used by the intelligent agent provides as follows. First, we divide the data set into *R* classes. Then we compute the distance between any two classes of patterns chosen from the *R* classes using an agent. We repeat it for all pair of such class patterns, where the distance between two classes is computed using the Minkowski distance. According this method, the distance between two points

(2) $$\begin{array}{c} P=\left(x_{1},x_{2},\ldots ,x_{n}\right),\;\;Q=\left(y_{1},y_{2},\ldots ,y_{n}\right)\in R^{n}. \end{array}$$

Now, the agent finds the center point of every class by using the formula,

(3) $$\begin{array}{c} \;{\left(\sum_{i=1}^{n}{\left|x_{i}-y_{i}\right|}^{p}\right)}^{1/p}, \end{array}$$

where *p* is the order and it also find the centroids of each class. 

The centroid is computed using the formula

(4) $$\begin{array}{c} C_{i}=\sum_{m=1}^{nt}\frac{X_{m}^{i}}{n_{i}}, \end{array}$$

where *C*_*i*_ = centroid value of *i*th node, *X* = individual *i*th lowest distance, *n* = number of dimensions.

Centroid of each class is found to compute the similarities with reference to the data value present in the dataset. After this calculation, the *k* classes obtained earlier are converted into *p* classes, where *p* is an optimum value selected by the agent. For example, when we considered a problem with 19 patterns, we obtained 10 classes after reduction. These 10 classes namely A, B, C, D, E, F, G, H, I, and J have been divided into 5 pairs namely AB, CD, EF, GH, and IJ. If the Minkowski distance of any two classes are less than that of the other classes then that pair is replaced by 1(Normal). Otherwise, it is replaced by −1 (Attacker). So, at end of the repeated process, we have only 1's and −1's combinations. Since −1 classes are removed, the remaining classes are used to construct the tree.

**Intelligent Agent-Based Enhanced Multiclass Support Vector Machine Algorithm (IAEMSVM)**

 
*Step  1.* Confirm two initial cluster centers by controlling the intelligent agents present in the sites.
 
*Step  2.* Import a new class *C*.
 
*Step  3.* Intelligent agent computes the Minkowski distance between two classes.
 
*Step  4.* If (d*AB* > *dAC*) then
      
*B* is assigned as normal.
   Else
      
*C* is assigned as attacker.
 
*Step  5.* Intelligent agent calculates the minimum and maximum of the distance.
 
*Step  6.* If (*d* *AB* < threshold limit of the distance) then create a new cluster and this is the center of the new cluster.
   Else
    
*B* is assigned as an Attacker.
 
*Step  7.* The intelligent agent repeats the operation until reduced the difference between the classes.

In this algorithm, intelligent agent helps to achieve better accuracy than the existing algorithms such as SVM, MSVM, and EMSVM. Moreover, this algorithm is used to reduce the execution time for using Minkowski distance measurement formula. Intelligent agent helps to take a better decision to classify the data through binary decision tree.

### 5.3. Weighted Outlier Detection

In this work, we propose an intelligent agent-based weighted distance algorithm based on the outlier factor to determine the outlierness algorithm of a point in the feature set in order to improve the detection accuracy. This proposed algorithm uses the relative location of a point with some assigned weight according to its perceived importance and its neighbor in the feature set to determine outlier of a point with respect to all clusters.

The formal definitions of weighted-distance-based outlier factor is presented as follows:

Let *s*_1_, *s*_2_,…, *s*_*k*_ be the *K*-nearest neighbors of an object *x* with weights *w*_1_, *w*_2_, *w*_3_,…, *w*_*k*_. The weighted distance of these *k*-nearest neighbors the object is defined as *w*_1_ *d*_1_, *w*_2_ *d*_2_,…, *w*_*k*_ *d*_*k*_ where *d*_1_, *d*_2_,…, *d*_*k*_ are the normal Euclidean distances *d*(*x*, *s*_*i*_) = *w*_*i*_ *d*_*i*_.

Their average weighted distance is computed using the formula

(5) 1k   ∗(∑i=1kwidi∑wi).

**Intelligent Agent-Based Weighted Distance Based Outlier Detection Algorithm (IAWDBOD)**

*Input.* The training Data Set.

*Output.* The set of *P* values when TS is two classes data set normal (*n*) and abnormal (*a*).

The intelligent agents are used to perform the training and testing phases given below effectively.

**Phase 1 : (Training)**

The outlier detection agents consists of four agents, namely, training agent, computation agent, decision agent, and testing agents

1. The training agent initiates the training and invokes the computation agent. The intelligent computation agent calculates the weighted average distance using (5).
2. The intelligent computation agent computes the number of nodes *n* whose distance is greater than the weighted average.
3. The intelligent computation agent computes the inner weighted average for the *k*-nearest inner nodes.
4. The training agent trains the data for inner and outer nodes by using the decision provided by the decision agent.

**Phase 2 : (Testing)**

The testing agent performs testing as follows.

1. The testing agent computes the new arriving node weighted distance using the weights assigned by the weight assignment agent.
2. If the distance of newly arrived node >the weighted average distance then the testing agent provides the result as abnormal else normal.

## 6. Exprementation and Results

### 6.1. Training and Testing Data

The dataset used in the experiment was taken from the Third International Knowledge Discovery and Data Mining Tools Competition (KDD Cup 99) [18]. Each connection record is described by 41 attributes. The list of attributes consists of both continuous type and discrete type variables, with statistical distributions varying drastically from each other, which makes the intrusion detection a very challenging task.

In this dataset, it has five million network connection records such as land attack, Neptune attack, password guess, and port scan. The 22 categories of attacks from the following four classes: DoS, R2L, U2R, and Probe. These 41 features describe the basic information about the network packet, network traffic, host traffic, and content information.  Table 1 shows the name and serial number of the 41 features. Each record contains the five class labels such as normal, probe, DOS, R2L, and U2R. It has 391458 DOS attack records, 52 U2R attack records, 4107 Probe attack record, 1126 R2L attack records, and 97278 normal records only in this 10 percent of this data set.

### 6.2. Experimental Results

The agent based attribute selection algorithm has selected 19 important features from 41 features in the dataset as shown in Table 2. This selection was based on the information gain ratio values of various attributes.

Table 3 shows the detection accuracies obtained using the 41 features of the KDD cup data set by applying the enhanced MSVM, WDBOD, and IAFSHC techniques. From this table, it can be seen that the proposed IAFSHC technique provides better detection accuracy for probe, DoS, and other attacks. This is due to the fact that this hybrid model makes use of the capabilities of intelligent agents for decision making and also makes use of the advantages of both the enhanced MSVM and WDBOD algorithms in which the first level decision is made using the enhanced MSVM algorithm where kernel function is used for effective classification.

On the other hand, this decision is further improved by applying weights to find the outliers. The combination of these two algorithms provides better results in all the experiments conducted in this research work. The experiments were conducted with 10 percentage of the KDD cup data set. However, each experiment used at least five different set of data records from the KDD cup data set and the average values were considered for each experiment.

Table 4 shows the detection accuracies of three classification techniques, namely, EMSVM, WDBOD, and IAFSHC when classification was performed using the 19 features which were selected by the feature selection algorithm. Moreover, it can be observed from Table 4 that the proposed IAFSHC technique provides better detection accuracy than the other two algorithms for all types of attacks, namely, Probe, DoS, and others. This is due to the fact that when all attributes were used in the decision making process, redundant values made confusions to the classifier. However, such confusions are resolved by calling the services of intelligent agents for decision making. Therefore, the number of iterations was increasing leading to increase in time. In addition to that the detection accuracy was less when they were compared with the accuracy obtained by applying the proposed IAFSHC with the selected 19 features.

Table 5 shows the performance analysis for the EMSVM algorithm and the proposed IAEMSVM algorithm based on training and testing time. From this analysis, it has been observed that the training and testing times are reduced in the proposed IAEMSVM algorithm when it is compared with EMSVM algorithm for Probe and DoS attacks.

Table 6 shows the performance comparison of WDBOD and the proposed IAWDBOD algorithm based on training and testing time. From this analysis, it has been observed that the training and testing times are reduced in the proposed IAWDBOD algorithm when it is compared with EMSVM algorithm for Probe and DoS attacks.

Table 7 shows the performance analysis in terms of training time, testing time, and classification accuracy for the proposed IAASA. From this table, it can be observed that the classification accuracy is increased for all types of attacks. Moreover, the training and testing times are reduced for the all the types of attacks, namely, probe, DoS, and others. Finally, this algorithm has selected 19 important features from 41 features of the given 10% of KDD cup data set. The accuracy improvement is achieved because the attributes which contribute correctly to the decision making process only are considered. Hence, the attributes that may confuse the classifier are eliminated. This leads to increase in accuracy. The decision accuracy is further improved by the rules derived and applied by the intelligent agent which is able to sense the environment to dynamically evolve the rules.

Table 8 shows the performance analysis for the proposed IAFSHC model which is developed by the combination of IAASA, EMSVM, and WDBOD. From this table, it can be observed that the detection accuracy is increased for all types of attacks. Moreover, the training and testing times for the all the types of attacks, namely, probe, DoS, and others are reduced in the proposed model. This is due to the fact that this proposed model considered only 19 features for classification. When the numbers of features are reduced, the classification needs less number of combinations leading to reduction in time.

**Figure 2:**
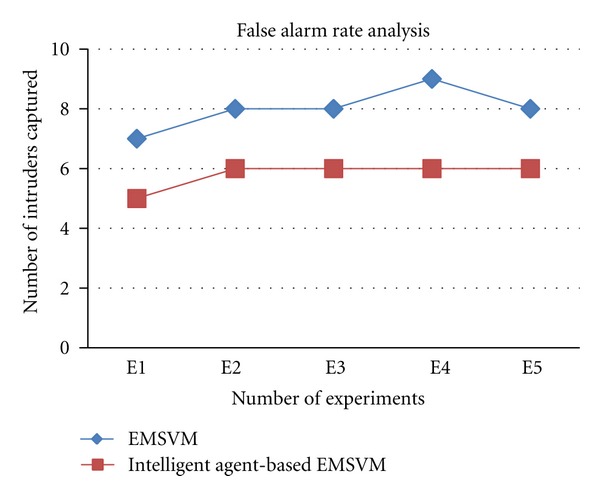
False alarm rate analyses for IAEMSVM.

Figure 2 shows the false alarm rate analysis for EMSVM and intelligent agent-based EMSVM (IAEMSVM). From the experiments conducted in this work, it is observed that the false alarm rate is low for intelligent agent-based EMSVM when it is compared with EMSVM. This improvement is because of the ability of the intelligent agents in making effective decision on choosing the correct metrics and rules for classification. 

**Figure 3:**
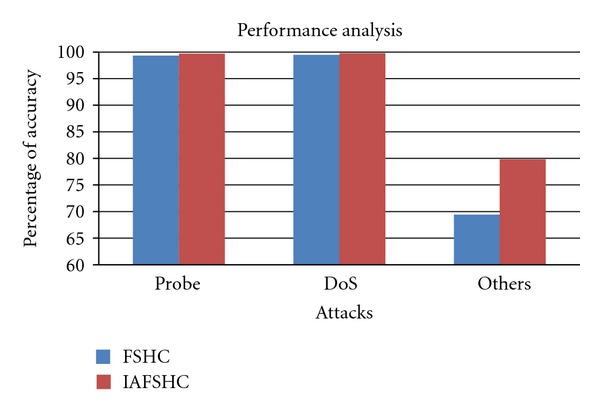
Results comparison between FSHC and IAFSHC.

Figure 3 provides the comparison of detection accuracies for DoS, Probe, and other attacks for the FSHC model and the proposed model IAFSHC. From this figure, it is observed that the proposed IAFSHC model provides better detection accuracy than the existing FSHC model. 

**Figure 4:**
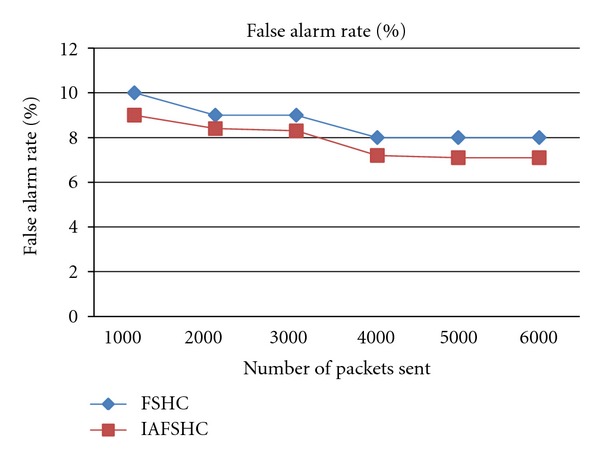
False alarm rate analysis for proposed Method.

Figure 4 shows the false alarm rate comparison between the proposed IAFSHC model and the existing FSHC model. From this figure, it is observed that the false alarm rate is reduced in the proposed IAFSHC model when it is compared with the FSHC model. This is due to the fact that in the proposed model classification accuracy was improved by introducing intelligent agents. 

From all these results, it can be observed that the proposed intrusion detection model reduced the false alarm rate and also the detection time when it is compared with the existing FSHC model. 

From Table 9, it can be observed that IAWDBOD with feature selection provides better accuracy when it is compared with IAWDBOD without feature selection. This is due to the fact that feature selection resolves the decision conflicts well. Second, the proposed IAEMSVM with feature selection provides better accuracy than the IAEMSVM without feature selection. This is not only because of efficiency of MSVM-based classification but also due to the clarity provided by the selected features. Third, the hybrid classifier with feature selection and without feature selection provides better classification accuracy due to the increase in decision parameters. Finally, the proposed intelligent agent-based feature selected hybrid classification model provides the highest accuracy when it is compared with all the other classification discussed in this table. This is due to the firing intelligent rules and the proper direction provided by the attribute selected by the feature selection method.

## 7. Conclusions and Future Works

In this paper, a new intrusion detection system called IAFSHC has been proposed and implemented for securing MANETs. This system has been developed by combining an intelligent agent-based weighted distance outlier detection (IAWDBOD) algorithm with another algorithm called intelligent agent-based enhanced multiclass support vector Machine (IAEMSVM) algorithm. Moreover, an effective preprocessing technique called intelligent agent-based attribute selection algorithm (IAASA) is proposed and included in IAFSHC to improve the detection accuracy and also to reduce the processing time. From the experiments conducted in this work, it has been observed that the classification accuracy for DoS, Probe, and other attacks are 99.77%, 99.70%, and 79.72%, respectively, when intelligent agents are added to the classifier. The main advantage of this method is that it reduces the false positive rates. Future works in this direction could be the use of fuzzy logic enhancing the power of the decision manager.

## Figures and Tables

**Table 1:** The 41 features in KDD Cup'99 Dataset.

S. No	Feature name	S. No	Feature name
1	duration	22	is_guest_login
2	protocol_type	23	count
3	service	24	serror_rate
4	src_byte	25	rerror_rate
5	dst_byte	26	same_srv_rate
6	flag	27	diff_srv_rate
7	land	28	srv_count
8	wrong_fragment	29	srv_serror_rate
9	urgent	30	srv_rerror_rate
10	hot	31	srv_diff_host_rate
11	num_failed_logins	32	dst_host_count
12	logged_in	33	dst_host_srv_count
13	num_compromised	34	dst_host_same_srv_count
14	root_shell	35	dst_host_diff_srv_count
15	su_attempted	36	dst_host_same_src_port_rate
16	num_root	37	dst_host_srv_diff_host_rate
17	num_file_creations	38	dst_host_serror_rate
18	num_shells	39	dst_host_srv_serror_rate
19	num_access_shells	40	dst_host_rerror_rate
20	num_outbound_cmds	41	dst_host_srv_rerror_rate
21	is_hot_login

**Table 2:** List of 19-selected features from 41 features.

S. No	Feature number	Feature name
1	2	protocol_type
2	4	src_byte
3	8	wrong_fragment
4	10	hot
5	14	root_shell
6	15	su_attempted
7	19	num_access_shells
8	25	rerror_rate
9	27	diff_srv_rate
10	29	srv_serror_rate
11	31	srv_diff_host_rate
12	32	dst_host_count
13	33	dst_host_srv_count
14	34	dst_host_same_srv_count
15	35	dst_host_diff_srv_count
16	36	dst_host_same_src_port_rate
17	37	dst_host_srv_diff_host_rate
18	38	dst_host_serror_rate
19	40	dst_host_rerror_rate

**Table 3:** Detection accuracy with 41 features.

Exp. number	Enhanced MSVM [5]			WDBOD [6]			IAFSHC		
	Probe	DoS	Others	Probe	DoS	Others	Probe	DoS	Others
1	99.00	99.12	69.19	99.49	99.62	70.52	99.53	99.51	69.60
2	98.90	99.12	68.91	99.39	99.22	68.72	99.10	99.17	69.38
3	98.92	99.02	69.10	99.51	99.41	73.32	99.63	99.52	69.73
4	99.10	99.12	69.19	99.24	99.14	72.25	99.62	99.58	69.79
5	99.15	99.07	68.89	99.32	99.13	70.92	99.54	99.49	69.19

**Table 4:** Detection accuracy with 19 features.

Exp. number	Enhanced MSVM [5]			WDBOD [6]			IAFSHC		
	Probe	DoS	Others	Probe	DoS	Others	Probe	DoS	Others
1	99.20	99.31	69.29	99.58	99.69	71.52	99.70	99.77	79.80
2	98.98	99.23	69.11	99.41	99.27	69.32	99.50	99.67	79.78
3	99.02	99.12	69.20	99.58	99.49	74.12	99.80	99.75	79.91
4	99.19	99.20	69.29	99.30	99.24	73.13	99.72	99.69	79.89
5	99.23	99.17	69.00	99.38	99.22	71.87	99.64	99.79	79.79

**Table 5:** Performance analysis for EMSVM and IAEMSVM.

Attacks	EMSVM		IAEMSVM	
	Training time (sec)	Testing time (sec)	Training time (sec)	Testing time (sec)
Probe	0.52	0.21	0.50	0.19
DoS	1.72	0.54	1.71	0.52
Others	0.53	0.17	0.51	0.15

**Table 6:** Performance analysis for WDBOD and IAWDBOD.

Attacks	WDBOD		IAWDBOD	
	Training time (sec)	Testing time (sec)	Training time (sec)	Testing time (sec)
Probe	0.62	0.22	0.60	0.20
DoS	1.92	0.64	1.88	0.62
Others	0.63	0.23	0.59	0.21

**Table 7:** Performance of the IAASA.

	Training time (sec)	Testing time (sec)	Accuracy (%)
Probe	0.42	0.21	99.77
DoS	1.76	0.84	99.87
Others	0.45	0.16	86.72

**Table 8:** Performance analysis for FSHC and IAFSHC.

Attacks	FSHC		IAFSHC	
	Training time (sec)	Testing time (sec)	Training time (sec)	Testing time (sec)
Probe	1.56	0.69	1.52	0.64
DoS	5.77	1.89	5.72	1.84
Others	1.65	0.63	1.61	0.57

**Table 9:** Performance analysis.

Exp. number	Overall detection accuracy (%)					
	IAWDBOD without feature selection	IAWDBOD with feature selection	IAEMSVM without feature selection	IAEMSVM with feature selection	IAHC	IAFSHC
1	89.95	90.33	89.05	90.37	89.49	93.08
2	89.23	90.23	88.91	90.31	89.12	93.12
3	89.92	89.92	89.02	89.27	88.91	94.01
4	89.72	90.42	89.12	89.92	89.54	92.45
5	88.97	90.37	89.01	90.32	89.20	92.92

Avg	89.56	90.25	89.02	90.04	89.25	93.12
